# Machine Learning and Deep Learning Models for Nocturnal High- and Low-Glucose Prediction in Adults with Type 1 Diabetes

**DOI:** 10.3390/diagnostics14070740

**Published:** 2024-03-30

**Authors:** Roman M. Kozinetz, Vladimir B. Berikov, Julia F. Semenova, Vadim V. Klimontov

**Affiliations:** Laboratory of Endocrinology, Research Institute of Clinical and Experimental Lymphology—Branch of the Institute of Cytology and Genetics, Siberian Branch of Russian Academy of Sciences (RICEL–Branch of IC&G SB RAS), 630060 Novosibirsk, Russia; romanec1954@gmail.com (R.M.K.); berikov@math.nsc.ru (V.B.B.); ekmxtyjr@yandex.ru (J.F.S.)

**Keywords:** type 1 diabetes, continuous glucose monitoring, glucose range, prediction, machine learning, deep learning, neural networks, random forest, boosting trees

## Abstract

Glucose management at night is a major challenge for people with type 1 diabetes (T1D), especially for those managed with multiple daily injections (MDIs). In this study, we developed machine learning (ML) and deep learning (DL) models to predict nocturnal glucose within the target range (3.9–10 mmol/L), above the target range, and below the target range in subjects with T1D managed with MDIs. The models were trained and tested on continuous glucose monitoring data obtained from 380 subjects with T1D. Two DL algorithms—multi-layer perceptron (MLP) and a convolutional neural network (CNN)—as well as two classic ML algorithms, random forest (RF) and gradient boosting trees (GBTs), were applied. The resulting models based on the DL and ML algorithms demonstrated high and similar accuracy in predicting target glucose (F1 metric: 96–98%) and above-target glucose (F1: 93–97%) within a 30 min prediction horizon. Model performance was poorer when predicting low glucose (F1: 80–86%). MLP provided the highest accuracy in low-glucose prediction. The results indicate that both DL (MLP, CNN) and ML (RF, GBTs) algorithms operating CGM data can be used for the simultaneous prediction of nocturnal glucose values within the target, above-target, and below-target ranges in people with T1D managed with MDIs.

## 1. Introduction

Glucose management at night is a major challenge for people with diabetes and clinicians. Real-world population-based studies showed a high incidence of nocturnal hypoglycemia, with reported rates of 2.6–11.3 events per patient-year in insulin-treated subjects with type 1 diabetes (T1D) [1]. The risk of the event appears to be trending downward as more patients receive continuous subcutaneous insulin infusion instead of multiple daily injections (MDIs) [2,3]. The introduction of sensor-augmented pumps with predictive low-glucose suspend and, especially, automated insulin delivery systems is a promising approach to further reduce the risk of hypoglycemia in T1D [4,5]. However, a significant proportion of patients are still managed with MDIs. There are a variety of nocturnal glucose profiles in these subjects. The patterns differ in bedtime and early morning glucose levels, the presence of downward and upward trends, and episodes of hypoglycemia; a lot of patients experience both hyperglycemia and hypoglycemia during the night [6]. Therefore, new technological solutions are urgently needed to predict glucose in patients managed with MDIs.

Continuous glucose monitoring (CGM) and machine learning (ML) have opened up new possibilities in glucose prediction. In recent years, various ML algorithms, including deep learning (DL), trained on CGM data or combinations of CGM data with other parameters were invented for this task. The results have been summarized in recent reviews [7,8,9] and meta-analyses [10]. Some studies were focused on nocturnal hypoglycemia specifically [11,12,13,14,15,16,17]. Taken together, the evidence indicates that data-driven models based on ML and DL algorithms have great potential in predicting glucose levels and hypoglycemic events. Most studies aim for short-term hypoglycemia prediction with a prediction horizon (PH) of 15 to 60 minutes [8]. However, attempts are being made to create hypoglycemia forecasting models with a longer PH [16,18].

To date, a number of ML algorithms have been tested for glucose prediction, including RF [12,13,15,16,17,19], artificial neural networks [15,20], support vector machines [11,12,13,15,17], linear discriminant analysis [14], logistic linear regression with Lasso regularization [15], and others. Recently, some models based on DL techniques have also been applied. Song et al. developed a method of forecast that combines empirical mode decomposition with long short-term memory (LSTM) [21]. Jaloli and Cescon proposed stacks of convolutional neural networks (CNNs) and LSTM units to predict glucose levels, taking into account historical glucose data, meal information, and insulin intakes [22]. Zhu et al. introduced a DL model based on a dilated recurrent neural network and, later, a fast-adaptive and confident neural network for glucose prediction in patients with T1D [23,24]. Dudukcu et al. applied LSTM, Wavenet, and gated recurrent units, as well as decision-level combinations of these architectures [25]. Van Doorn et al., using a large-scale diabetes dataset, demonstrated that DL-based models provide accurate glucose prediction in both type 1 and type 2 diabetes. In their study, a classical recurrent neuron network architecture had superior performance within a 15-minute PH, while an LSTM network outperformed all other algorithms within a 60-minute PH [26]. An approach with a stacked LSTM-based deep recurrent neural network model with the Kalman smoothing technique for the correction of inaccurate CGM readings was introduced by Rabby et al. [27]. The results of these studies suggest the superiority of DL approaches over traditional ML algorithms in glucose prediction. Advanced glucose prediction models based on CGM data and ML or DL algorithms are generally considered to be promising elements for closed-loop automatic insulin delivery systems. Zafar et al. successfully incorporated ML-based and DL-based methods of glucose prediction in individuals with open-source automated insulin delivery systems [28]. 

In recent years, the concept of timing in glucose ranges has entered diabetes management. Time in range generally refers to the time spent in an individual’s target glucose range (usually 3.9–10 mmol/L, or 70–180 mg/dL [29]). Other widely accepted metrics are time above target glucose range and time below target glucose range [30]. Accumulating evidence suggests that time in range is a predictor of vascular morbidity and mortality in people with diabetes [31,32,33]. Currently, models for the prediction of glucose values in the above-mentioned glycemic ranges in people with diabetes managed with MDIs have not yet been developed. Such models could be used to increase the time in range, prevent excessive glucose fluctuations, and reduce glucose variability, another established risk factor for diabetic complications [34,35].

In this study, we aimed to develop ML-based and DL-based models to predict glucose levels within the target range (3.9–10 mmol/L, or 70–180 mg/dL), above the target range (>10 mmol/L, or >180 mg/dL), and below the target range (<10 mmol/L, or <70 mg/dL) in patients with T1D managed with MDIs. To our knowledge, this is the first study focused on this task specifically. For the model generations, we used CGM data of real patients with T1D. Two DL algorithms, multi-layer perceptron (MLP) and CNNs, and two ML algorithms, random forest (RF) and gradient boosting trees (GBTs), were applied for the model generation. We hypothesized that DL algorithms can outperform ML techniques in prediction accuracy.

The results indicate that both DL (MLP, CNNs) and ML (RF, GBTs) algorithms trained on CGM data can provide high accuracy when predicting glucose levels within the target range and above the target range within a 30-minute PH. However, predicting glucose within the range below the target (<3.9 mmol/L, or <70 mg/dL) proved to be a more difficult challenge. In this case, MLP slightly outperformed the other models.

## 2. Materials and Methods

### 2.1. Database

For the model generation, we used a database of CGM recordings from the RICEL–branch of IC&G SB RAS, a tertiary referral hospital. This database was registered by the Federal Service for Intellectual Property (Rospatent; certificate 2023623235 dated 26 September 2023). Data from 406 adult individuals with T1D managed with MDIs were selected. To make the sample more homogeneous, data from patients in a current diabetic ketoacidosis or hyperglycemic hyperosmolar state, as well as those with end-stage renal disease, acute infections, and severe accompanying diseases, were not included.

All patients underwent an assessment of metabolic control and diabetic complications at the hospital in accordance with current national guidelines. Blinded CGM was performed in hospital settings with an iPro™2, MMT-7741 CGM system and CareLink iPro™ (CareLink iPro, MMT-7740) software (https://carelink.minimed.eu/ipro/hcp/index.jsp (accessed on 26 December 2023)) (Medtronic, Minneapolis, MN, USA). The mean CGM duration was 6.7 days.

### 2.2. Data Preprocessing

Overnight intervals (0–6 a.m.) of interstitial glucose measurements were used for analysis. The CGM data were presented as time series of glucose levels with up to 72 values in each interval. Records with missing values were excluded. The refined dataset included information from 380 subjects.

### 2.3. Modeling

Each time series was divided into overlapping subsequences of a given length (size of lookback window, LBW) with successive starting points. Each subsequence was used by a model to predict glucose levels within the target range, above the target range, and below the target range. Thus, we considered a time-series classification task with three prediction classes. The target glucose range was defined as 3.9–10 mmol/L, or 70–180 mg/dL, according to the International Consensus on the Use of CGM [29].

In this study, we used two DL algorithms (MLP and CNNs). Since deep neural nets can use raw data and extract features automatically, we used glucose levels in a series as input features for each model. Thus, no feature engineering stage was involved in this analysis.

We also built models based on two ML algorithms (RF, GBTs) and compared their performance with that of the DL models.

#### 2.3.1. MLP

**Figure 1:**
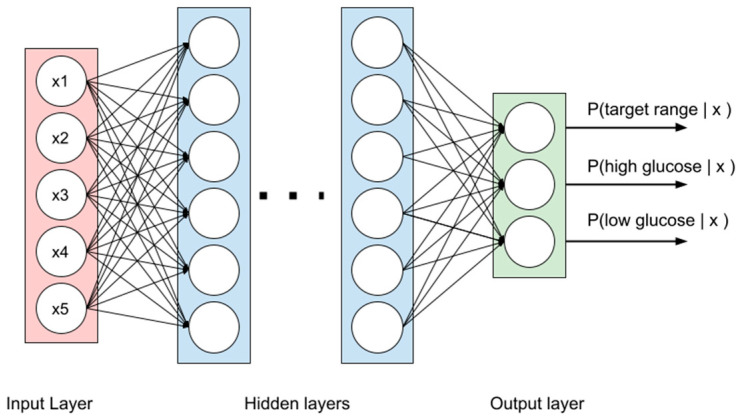
An example of MLP architecture.

We used several variants of MLP architecture to estimate the optimal network depth. An example of MLP architecture is shown in Figure 1, and the detailed description of the considered architectures is presented in Table 1.

All neural networks were trained on 40 epochs with the Stochastic Gradient Descent optimizer, with a batch size of 64, a momentum of 0.9, a learning rate scheduler with step of 10, and a gamma of 0.1.

#### 2.3.2. CNNs

**Figure 2:**
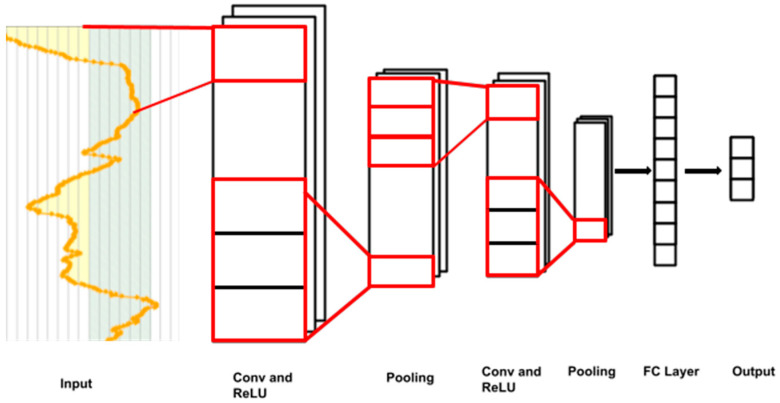
Convolution of signals throughout a network.

We applied a one-dimensional variant of CNNs, where one-dimensional transformations were used as the convolutions (Figure 2). As in the case of MLP, we studied several CNN models with different depth levels. The details of the CNN architectures are provided in Table 2.

Input fragments are convoluted to new subsequences. Different convolution filters produce different output channels.

#### 2.3.3. RF and GBTs

Two models based on the RF and GBTs were built to match the results with those of deep learning models. We selected an optimal number of trees for RF and GBTs using cross-validation with 5 folds. B = 160 was chosen for RF and M = 100 for GBTs. The maximum depth of a tree was 5 for RF and 3 for GBTs. For GBTs, we used a learning rate of 0.1. The default values for the rest of the hyperparameters were taken.

We implemented the used models as a program code written in Python 3.8 on top of the PyTorch and Scikit-Learn open-source libraries.

### 2.4. Evaluation of the Models

The data were randomly divided into 80% of the participants for training and 20% for evaluation. After excluding inappropriate records with missing values, data from 306 patients were included in a training set and data from 74 people in a test set. The training set included 81,749 glucose values within the target range, 33,310 values within the range above the target, and 5389 values within the range below the target. The test set comprised 29,800 glucose values within the target range, 8087 values in the range above the target, and 1205 values within the range below the target.

Taking into account the different number of observations in the three ranges, we applied data balancing techniques for the training dataset. After undersampling and oversampling had been tested, we chose the undersampling procedure, as it gave better results.

Since we were solving the problem of classifying glucose values into the mentioned ranges, and taking into account the imbalance of the data, Precision, Recall, and F1 metrics were chosen to evaluate the performance of the models (Table 3). These metrics were evaluated for the optimal decision threshold obtained from the averaged receiver operating characteristic–area under the curve (ROC-AUC).

## 3. Results

In this section, we present clinical characteristics of patients, performance metrics of the DL and ML models, and data on the effect of PH and LBW length on the accuracy of glucose prediction.

### 3.1. Characteristics of Patients

In total, we analyzed data from 380 patients with T1D, 138 men and 242 women, aged from 18 to 67 years (median: 36 years). The duration of diabetes ranged from 0.5 to 55 years (median: 16 years). The mean level of hemoglobin A1c (HbA1c) was 8.1% or 64.8 mmol/mol (range: 4.7–15.1% or 27.9–141.8 mmol/mol). All patients were managed with MDIs of long-acting and short-acting insulin analogues. The daily insulin dose was 0.66 IU/kg (range: 0.2–2.0 IU/kg).

The clinical characteristics of patients in the training and test samples are presented in Table 4. These samples did not differ in gender, age, anthropometric, clinical, and metabolic characteristics of the participants.

### 3.2. Performance Metrics of the Models

The metrics of the built models for glucose prediction at 30 min PH and 30 min LBW are presented in Table 5.

All of the algorithms we used performed well when predicting glucose levels within the target range and within the range above the target. Thus, the values of the F1 metric varied from 96 to 98% when predicting target glucose levels and from 93 to 97% when predicting above-target glucose. For the target and above-target glucose ranges, all tested algorithms showed almost identical and very high Precision, Recall, and F1 values.

When predicting glucose within the range below the target, the Precision, Recall, and F1 metrics were lower for all tested models (F1 values varied from 83 to 86%). For this range, the GBT model provided the highest Recall value, but the integral F1 metrics were the highest in the MLP-based models.

### 3.3. Effects of PH and LBW on the Model Performance

We evaluated effects of PH and LBW length on model performance. CNN4 and the GBTs were chosen as examples of DL and ML models, respectively. The F1 metrics averaged over the glucose ranges at different PH and LBW lengths are presented in Table 6.

CNN4 slightly outperformed the GBTs (by about 1% for F1 metric when averaged over PH and LBW values). The effect of the LBW size was also not substantial in both models. As the length of the PH increased, the quality of the models decreased significantly.

## 4. Discussion

### 4.1. Methodology and Principal Results of This Study

The elaboration of reliable methods of glucose forecasting is essential for improving glycemic control in people with diabetes. In this study, we aimed to develop CGM-based ML and DL models for the short-term prediction of nocturnal glucose levels within the target range, above-target range, and below-target range in patients with T1D managed with MDIs. We studied a number of different network architectures and evaluated their performance on the test sample. The models were trained and tested on real CGM data derived from 380 T1D patients, representing a wide range of glycemic patterns and clinical profiles.

To generate the models, we applied two DL algorithms, MLP and CNNs. In the MLP, there are several layers of neurons; each neuron is connected with all the others in the next layer. The network structure includes the input layer, taking the values of glucose levels; hidden layers, performing a combination of linear operations over inputs with non-linear activations; and the output layer, yielding the probability distribution of classes. The weights used in linear operations are learned during model training [37]. CNNs are based on the use of the convolution operation, and they are formed by a locally connected network with successive convolutional layers. These networks have fewer weights than MLP and preserve the spatial information of the original data [38]. The results obtained were compared with those of models based on classical ML techniques: RF and GBTs. The RF is an ensemble of decision trees, where each tree is trained on a random subset of the data. During prediction, the final output is determined by averaging or voting over the individual trees’ predictions, leading to improved accuracy and robustness against overfitting [39]. GBTs build an ensemble in an adaptive manner, where each new tree corrects the errors made by the previous ones. They use gradient descent optimization techniques to minimize the loss function, improving model performance by focusing on misclassified instances [40].

To assess the quality of the models, we applied the Precision, Recall, and F1 metrics to reflect the balance between true-positive, false-positive, and false-negative outcomes. The models based on the DL and ML techniques demonstrated comparable accuracy. At 30-minute PH and 30-minute LBW lengths, all applied algorithms performed well when predicting glucose values in the target range (3.9–10 mmol/L, or 70–180 mg/dL; F1 metric 96–98%) and glucose values in the range above the target (>10 mmol/L, or >180 mg/dL; F1 metric 93–97%). However, the forecasting of low glucose values (<3.9 mmol/L, or <70 mg/dL) turned out to be a more difficult challenge (F1 metric values varied from 80 to 86%). This may be explained by fewer glucose values in this range. By definition, the range below the target is narrower than the other two ranges. Moreover, this range is artificially reduced by the detection limit of CGM systems (2.2 mmol/L for the CGM systems we applied in this study). It is possible that the use of large datasets can overcome this limitation. Regarding the algorithms, MLP slightly outperformed the other models in predicting glucose levels within the range below the target.

Selecting the most appropriate PH is a very important step in ML. In relation to the situation under discussion, on the one hand, it is important to predict episodes of high and low glucose as far in advance as possible. On the other hand, an increase in the PH length usually leads to deterioration in the quality of the forecast [26]. We compared the performance of our models at different PHs and LBWs. Among the top-ranking models, deep CNNs slightly outperformed GBTs at a PH of 15, 30, 45, 60, and 75 minutes (Table 5). Meanwhile, elongation of the LBW from 15 to 75 minutes did not significantly affect the classification performance. This can be explained by the fact that the most important predictive information is spread over the most recent glucose measurements. Expectedly, as the length of the PH increased, the quality of the models decreased significantly. We believe that 30-minute PH is the optimal compromise between the need to have time to prevent an adverse event and the reliability of the forecast. This PH has been selected in many studies focused on ML-based glucose prediction [8].

### 4.2. Comparisons with Other Studies

By now, a number of studies have addressed the problem of predicting glucose levels in patients with diabetes with the use of ML or DL techniques. These studies are quite different methodologically. In many studies, CGM data were obtained from patients on continuous subcutaneous insulin infusion. Only a few studies used data from patients on MDIs to train the models [11,14,16].

In many studies, the authors focused on forecasting events such as nocturnal hypoglycemia [11,12,13,14,15,16,17]. Other studies predicted interstitial glucose values [21,22,23,24,25,26,27,28]. In this study, we proposed a different approach for glucose prediction by classifying the predicted values into three ranges. To our knowledge, this is the first time this approach has been implemented. In our opinion, the advantage of this approach is the ability to tune the model for use in a specific glucose range. Previously, Guemes et al., using ML algorithms for binary classification and the OhioT1DM dataset, proposed an approach to predict whether overnight blood glucose concentrations would remain within or outside the target range [41].

Most studies on the use of ML or DL techniques achieved fairly high predictive accuracy. In studies forecasting nocturnal hypoglycemia, the values of ROC-AUC exceeded 70%, indicating an acceptable sensitivity and specificity [11,12,13,14,15,16,17]. In studies predicting glucose levels, the root mean squared error varied from 0.36 to 1.95 mmol/L (6.45–35.10 mg/dL) at PH values up to 120 minutes [21,22,23,24,25,26,27,28]. In the aforementioned study by Guemes et al., which addressed the problem of classifying future glucose levels into the target and non-target ranges, the model was able to predict the quality of overnight glycemic control with reasonable accuracy (AUC–ROC = 0.7) [41]. In our study, we achieved very high accuracy in predicting glucose levels in the target and above-target ranges when evaluating models based on the Precision, Recall, and F1 metrics. Glucose prediction in the below-target range proved to be a more difficult task. However, even in this case, the metric values were in the range of 74–94%. This allows us to consider our results as potentially acceptable from a clinical point of view.

In diabetes management, glucose prediction models can be incorporated into mobile applications and automated insulin delivery systems. We believe that our approach to glucose range prediction may be more appropriate for patients managed with MDIs, whereas approaches focused on predicting specific glucose values are more relevant for closed-loop automated insulin delivery systems.

### 4.3. Limitations of This Study and Future Remarks

The recruitment of patients in one clinical center, the relatively small sample size, and the short CGM duration are obvious limitations of our study. The models were based on CGM data exclusively and did not take into account any behavioral and clinical parameters of the participants. Finally, we did not validate our models on any external datasets.

Studies with greater statistical power are needed to develop more reliable models for low-glucose prediction. The models that predict glucose within the ranges can be used in mobile applications for people with diabetes. The evaluation of the clinical effectiveness of such applications in preventing nocturnal episodes of hyperglycemia and hypoglycemia is a challenge for future research.

## 5. Conclusions

In this study, we proposed a new approach for nighttime glucose prediction in T1D patients managed with MDIs based on CGM data and ML or DL algorithms. This approach involves classifying future glucose values into the target range (3.9–10 mmol/L, or 70–180 mg/dL), above-target range (>10 mmol/L, or >180 mg/dL), and below-target range (<3.9 mmol/L, or <70 mg/dL). For the model generations, we used two DL algorithms, MLP and CNNs, and two ML algorithms, RF and GBTs. The results indicate that both DL and ML models provide high accuracy when predicting glucose within the target range and the range above target within a 30-minute PH. The performance of the models in predicting glucose levels within the range below the target was slightly poorer, and MLP-based models showed the best performance here. The further introduction of mobile applications based on the developed models seems to be a promising approach to reduce the burden of both nocturnal hyperglycemia and hypoglycemia in subjects with T1D managed with MDIs.

## Figures and Tables

**Table 1:** The architectures of MLPs used in this study for glucose prediction.

Model	Layers	Layer Dimensions
MLP 1	Fully connected + ReLU	[n_input, 64]
	Fully connected + Softmax	[64, 3]
MLP 2	Fully connected + ReLU	[n_input, 64]
	Fully connected + ReLU	[64, 128]
	Fully connected + Softmax	[128, 3]
MLP 3	Fully connected + ReLU	[n_input, 64]
	Fully connected + ReLU	[64, 128]
	Fully connected + ReLU	[128, 128]
	Fully connected + Softmax	[128, 3]
MLP 4	Fully connected + ReLU	[n_input, 64]
	Fully connected + ReLU	[64, 128]
	Fully connected + ReLU	[128, 128]
	Fully connected + ReLU	[128, 64]
	Fully connected + Softmax	[64, 3]

Each variant of architecture is represented with a list of its layers and activation functions, as well as with its layer’s dimensionalities. MLP1, …, MLP4 are the different versions of a fully connected neural network with the ReLU [36] activation function between hidden layers and the Softmax activation function after the last output layer. Layer dimensions represent the number of input and output connections of each layer in the network. The parameter n_input denotes the number of neurons in the first layer; it equals the dimensionality of input data. In our study, n_input = LBW.

**Table 2:** The architectures of CNNs used in this study for glucose prediction.

CNN 1	CNN 2	CNN 3	CNN 4
Conv1d(1,32,3)	Conv1d(1,32,3)	Conv1d(1,32,3)	Conv1d(1,32,3)
BatchNorm	BatchNorm	BatchNorm	BatchNorm
ReLU	ReLU	ReLU	ReLU
Conv1d(8,16,3)	Conv1d(8,16,3)	Conv1d(8,16,3)	Conv1d(8,16,3)
BatchNorm	BatchNorm	BatchNorm	BatchNorm
ReLU	ReLU	ReLU	ReLU
Conv1d(16,32,3)	Conv1d(16,32,3)	Conv1d(16,32,3)	Conv1d(16,32,3)
BatchNorm	BatchNorm	BatchNorm	BatchNorm
ReLU	ReLU	ReLU	ReLU
Conv1d(32,64,3)	Conv1d(32,64,3)	Conv1d(32,64,3)	Conv1d(32,64,3)
BatchNorm	BatchNorm	BatchNorm	BatchNorm
ReLU	ReLU	ReLU	ReLU
AveragePooling1d	Conv1d(64,128,3)	Conv1d(64,128,3)	Conv1d(64,128,3)
	BatchNorm	BatchNorm	BatchNorm
	ReLU	ReLU	ReLU
Fully connected(64,3) + Softmax	AveragePooling1d	Conv1d(128,256,3)	Conv1d(128,256,3)
		BatchNorm	BatchNorm
		ReLU	ReLU
	Fully connected(128,3) + Softmax	AveragePooling1d	Conv1d(256,512,3) BatchNorm ReLU
		Fully connected(256,3) + Softmax	AveragePooling1d
			Fully connected(512,3) + Softmax

For each variant of the architecture, a list of its components is presented. CNN1, …, CNN4 are the different variants of the one-dimensional convolutional network. Each of them has several blocks of 1D convolution, a batch normalization layer, an ReLU activation function, and a Softmax activation function after the last output layer. Convolution operator Conv1d(cin,cout,k) includes numbers of input and output channels; k is a kernel size; BatchNorm is a normalization operator.

**Table 3:** Performance metrics used for the assessment of glucose prediction models.

Metric	Formula
Precision	$\frac{TP}{TP\;+\;FP}$
Recall	$\frac{TP}{TP\;+\;FN}$
F1	$\frac{2\;*\;Precision\;*\;Recall}{Precision\;+\;Recall}$

*TP*, *FP*, and *FN* denote true-positive, false-positive, and false-negative outcomes, respectively. For each of the three considered classes, these binary classification metrics were evaluated using the one-vs-rest method based on the confusion matrix obtained with a model.

**Table 4:** Clinical characteristics of T1D patients in the training and test samples.

Parameter	Training Sample (*N* = 306)	Test Sample (*N* = 74)	*p*
Sex, m/f, *n* (%)	108 (35.3)/198 (64.7)	30 (40.5)/44 (59.5)	0.40
Age, years	36 (27; 49)	36 (28; 50)	0.73
Body mass index, kg/m^2^	23.9 (21.4; 27.4)	23.3 (21.2; 25.9)	0.26
Diabetes duration, years	16 (10; 25)	15 (8; 28)	0.85
Insulin dose, IU/kg/day	0.7 (0.54; 0.83)	0.6 (0.5; 0.85)	0.42
Basal insulin dose, IU/kg/day	0.28 (0.21; 0.38)	0.25 (0.21; 0.33)	0.06
Diabetic retinopathy, *n* (%)	182 (59.5)	43 (56.3)	0.83
Chronic kidney disease, *n* (%)	206 (67.3)	52 (70.3)	0.63
Arterial hypertension, *n* (%)	118 (38.6)	35 (47.3)	0.17
Coronary artery disease, *n* (%)	23 (7.5)	5 (6.8)	0.82
Neuropathy, *n* (%)	205 (67)	49 (66.2)	0.9
Impaired awareness of hypoglycemia, *n* (%)	114 (37.3)	21 (28.4)	0.15
HbA1c, %	8.1 (7.1; 9.2)	7.7 (6.9; 8.9)	0.34
HbA1c, mmol/mol	64.8 (53.7; 76.5)	60.3 (52.2; 74.4)	0.34
Total cholesterol, mmol/L	5.0 (4.2; 5.9)	5.1 (4.4; 5.8)	0.91
Triglycerides, mmol/L	82 (73; 93)	79 (75; 95)	0.92
Serum creatinine, µmol/L	88 (72; 99)	85 (74; 97)	0.58
eGFR, mL/min/1.73 m^2^	0.5 (0.3; 1.1)	0.6 (0.3; 1.6)	0.85
UACR, mg/mmol	16 (10; 25)	15 (8; 28)	0.85

Continuous data are presented as medians (25th; 75th percentiles). eGFR, estimated glomerular filtration rate; HbA1c, glycated hemoglobin A1c; T1D, type 1 diabetes; UACR, urinary albumin-to-creatinine ratio.

**Table 5:** Performance metrics (%) of the DL and ML models for predicting interstitial glucose levels within the target range, above the target range, and below the target range at 30 min PH and 30 min LBW in patients with T1D managed with MDIs.

Model	Target Glucose Range (3.9–10 mmol/L, or 70–180 mg/dL)			Above Target Glucose Range (>10 mmol/L, or >180 mg/dL)			Below Target Glucose Range (<3.9 mmol/L, or <70 mg/dL)		
	Precision	Recall	F1	Precision	Recall	F1	Precision	Recall	F1
MLP 1	98	95	96	90	97	93	77	91	83
MLP 2	98	98	98	96	97	96	87	86	86
MLP 3	99	98	98	96	97	96	84	88	86
MLP 4	99	98	98	96	97	96	84	88	86
CNN 1	99	97	98	94	97	95	74	86	80
CNN 2	98	98	98	97	97	97	80	87	83
CNN 3	99	98	98	95	97	96	80	88	84
CNN 4	98	98	98	97	96	96	82	89	85
RF	99	97	98	97	97	97	82	88	85
GBTs	99	98	98	96	98	97	78	94	85

For each predicted range, Precision, Recall, and F1 metrics were evaluated using one-vs-rest technique. CNN1–CNN4, convolutional neural network 1–4; DL, deep learning; GBTs, gradient boosted trees; LBW, lookback window; MDIs, multiple daily insulin injections; ML, machine learning; MLP1–MLP4, multi-layer perceptron 1–4; PH, prediction horizon; RF, random forest; T1D, type 1 diabetes.

**Table 6:** F1 metrics (%) averaged over target, above-target, and below-target glucose ranges of CNN4-based and GBT-based glucose prediction models depending on PH and LBW length.

PH	15 min	30 min	45 min	60 min	75 min
CNN 4					
LBW = 15 min	97	93	90	87	85
LBW = 30 min	97	93	91	88	86
LBW = 45 min	97	93	90	88	86
LBW = 60 min	97	93	89	87	86
LBW = 75 min	97	93	90	87	86
GBTs					
LBW = 15 min	98	93	89	86	84
LBW = 30 min	97	92	89	87	85
LBW = 45 min	97	93	89	87	85
LBW = 60 min	97	92	89	86	85
LBW = 75 min	97	92	89	87	85

The averaging was performed over high, target, and low glucose levels. CNN4, convolutional neural network 4; GBTs, gradient boosted trees; LBW, lookback window; PH, prediction horizon.

## Data Availability

The source data are available from the corresponding author upon reasonable request.
